# Utility of Whole Body 18F-FDG PET/CT in Comparison to Pelvic MRI in Evaluation of Local Staging of Early-Stage Carcinoma Cervix

**DOI:** 10.7759/cureus.32111

**Published:** 2022-12-01

**Authors:** Nagesh Kumar Singaram, Narendra Hulikal, Bodagala Vijayalakshmi Devi, Ranadheer Manthri, Amith Kumar Chowhan

**Affiliations:** 1 Surgical Oncology, Sri Venkateswara Institute of Medical Sciences, Tirupati, IND; 2 Radiology, Sri Venkateswara Institute of Medical Sciences, Tirupati, IND; 3 Nuclear Medicine, Mehdi Nawaz Jung Institute of Oncology Regional Cancer Center, Hyderabad, IND; 4 Pathology, All India Institute of Medical Sciences, Raipur, IND

**Keywords:** figo 2009, lymph node, parametrium, mri, pet/-ct, early stage carcinoma cervix

## Abstract

Objective: This prospective comparative study aimed to investigate the applied value of whole body 2-deoxy-2[fluorine-18]fluoro- D-glucose positron emission tomography integrated with computed tomography (^18^F-FDG PET/CT) in comparison to pelvic magnetic resonance imaging (MRI) in early cervical cancer patients.

Material and methods: A prospective study was performed on 47 clinically early-stage cervical cancer patients evaluated with positron emission tomography/computed tomography (PET/CT) and MRI before surgery. The final postoperative histopathology report served as the reference standard. Both PET/CT and MRI images were analyzed and correlated with histopathologic findings concerning parametrial and lymph node involvement.

Results: Sensitivity, specificity, and negative predictive value (NPV) of PET/CT were 33.3%, 81.8%, and 94.7%, respectively, for parametrium assessment. And the corresponding values of pelvic MRI were 33.3%, 63.6%, and 93.3%, respectively (PET/CT versus MRI, p > 0.05). The positive predictive value (PPV) of PET/CT (11.1%) was higher than MRI (5.9%) for parametrial assessment (p < 0.05). The sensitivity, specificity, PPV, and NPV of PET/CT were 75%, 83.7%, 30%, and 97.3%, respectively, for lymph node assessment. And the corresponding values of MRI were 75%, 81.3%, 27.3%, and 97.2%, respectively (PET/CT versus MRI, p > 0.05). There was no significant difference between MRI and PET/CT concerning stage migration (p = 0.4276).

Conclusion: The PET/CT had no additional utility (compared to MRI) in the evaluation of local staging of clinically early cervical carcinoma patients.

## Introduction

Carcinoma cervix is the fourth most common malignancy among women in the world and is one of the leading causes of cancer-related mortality. The incidence of cervical cancer is 16.5% and has a mortality rate of 7.5% [1]. There is a significant survival difference between node-positive and negative cases (90% versus 50%). Therefore, accurate staging and detection of lymph node metastases are essential to ensure proper treatment planning and prediction of prognosis for cervical cancer patients [2].

Magnetic resonance imaging (MRI) and computerized tomography (CT) have been widely used to detect lymph node metastases, which are based on morphologic information. Whole body 2-deoxy-2[fluorine-18]fluoro- D-glucose positron emission tomography integrated with computed tomography (18F-FDG PET/CT) offers combined benefits of anatomic and functional imaging [3].

Surgical lymphadenectomy is the reference standard in the diagnosis of nodal metastases, but it is associated with complications. Therefore, preoperative imaging assessment of nodes is important [4].

With the use of appropriate imaging, accurate staging can be done which can change the treatment modalities, the extent of treatment, and adjuvant treatment. Though the individual role of MRI, positron emission tomography/computed tomography (PET/CT) in the staging of a cervical cancer patient is recognized, and the role of PET/CT in advanced carcinoma cervix is defined, there are very few prospective studies comparing MRI and PET/CT in the evaluation of early carcinoma cervix patients (International Federation of Gynaecology and Obstetrics (FIGO) stage I to IIA). Hence, the present study was planned to know the utility of 18F-FDG PET/CT in comparison to MRI in the evaluation of clinically operable patients with early carcinoma cervix.

The objectives of the present study were to compare the sensitivity, specificity, positive predictive value (PPV), and negative predictive value (NPV) of MRI and 18F-FDG PET/CT for the detection of local extension and lymph-nodal metastases and to analyze the percentage of cases undergoing stage migration with 18F-FDG PET/CT and MRI.

## Materials and methods

This prospective analytical study was conducted at Sri Venkateswara Institute of Medical Sciences University Teaching Hospital, Tirupati, Andhra Pradesh, India, from June 2018 to November 2019. Study participants were recruited after obtaining approval from the Institutional Ethics Committee (approval no.: AS/11/IEC/SVIMS/2017). Written informed consent was obtained from all the subjects participating in the study. Biopsy-proven carcinoma cervix patients of the age group 18 to 80 years; FIGO I/IIA (FIGO 2009); treatment-naive; those who gave informed consent and who underwent both PET/CT, MRI, and subsequent surgery were included. Patients who underwent surgery elsewhere; received prior radiation or chemotherapy; have recurrent cervix cancer, poor performance status, abnormal renal function tests, and contraindications for MRI; pregnant women and lactating mothers; and those not willing to take part in the study were excluded.

The FIGO (2009) staging was assigned to all the study participants based on clinical assessment by an experienced surgeon. Cervical punch biopsy was performed under local anesthesia five to seven days before MRI and only biopsy-proven patients were evaluated by MRI and PET/CT. The PET/CT and MRI reading physicians were blinded to the clinical details of the patient, other imaging findings, and final histopathological findings. All patients underwent modified radical/radical hysterectomy seven to 10 days after imaging, based on the FIGO (2009) staging. A senior pathologist did histopathology reporting.

**Figure 1:**
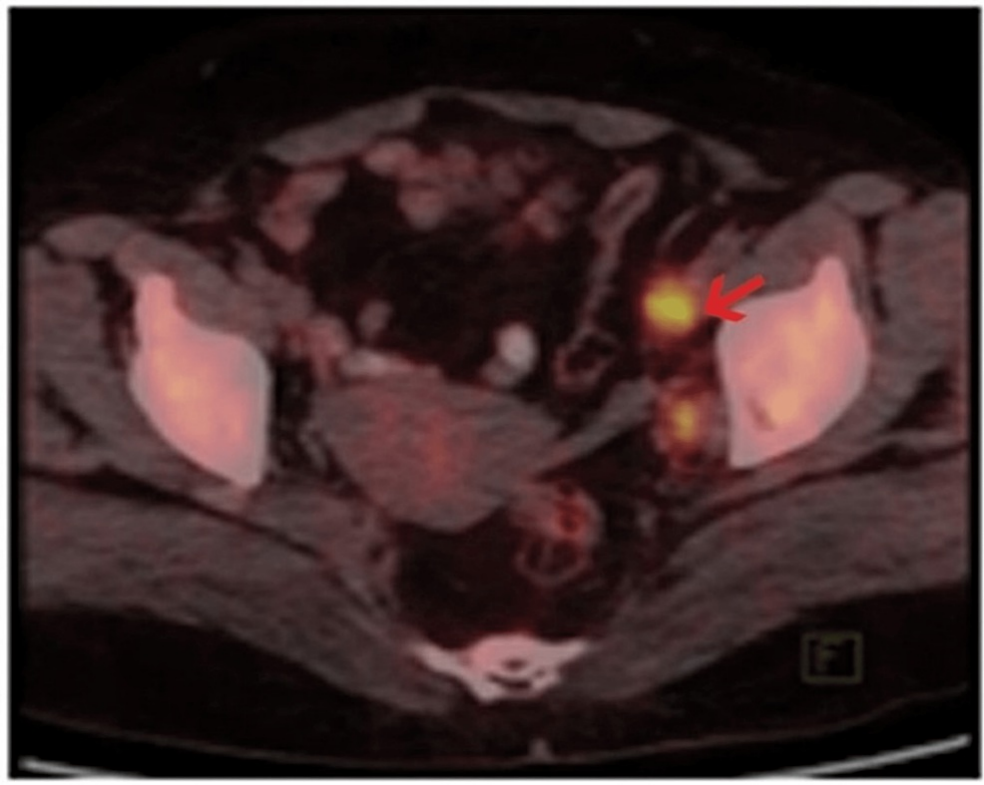
The PET/CT image showing left pelvic lymph node involvement The red arrow indicates FDG uptake of the left pelvic lymph node of an early cervical cancer patient. PET/CT: Positron emission tomography/computed tomography, FDG: Fluoro- D-glucose

Whole body 18F-FDG PET/CT (Biograph-6, lutetium oxyorthosilicate (LSO), PET/CT scanner by Siemens, Munich, Germany) was performed following standard operating procedure guidelines by the Society of Nuclear Medicine (SNM) and European Association of Nuclear Medicine (EANM). Images were analyzed and interpreted qualitatively using quantitative analysis of 18F-FDG uptake with maximum standardized uptake value (SUVmax) and retention index (RI). If FDG uptake of the lymph node (SUV) was more than the surrounding tissue, then it was considered as positive lymph nodal involvement as shown in Figure 1.

**Figure 2:**
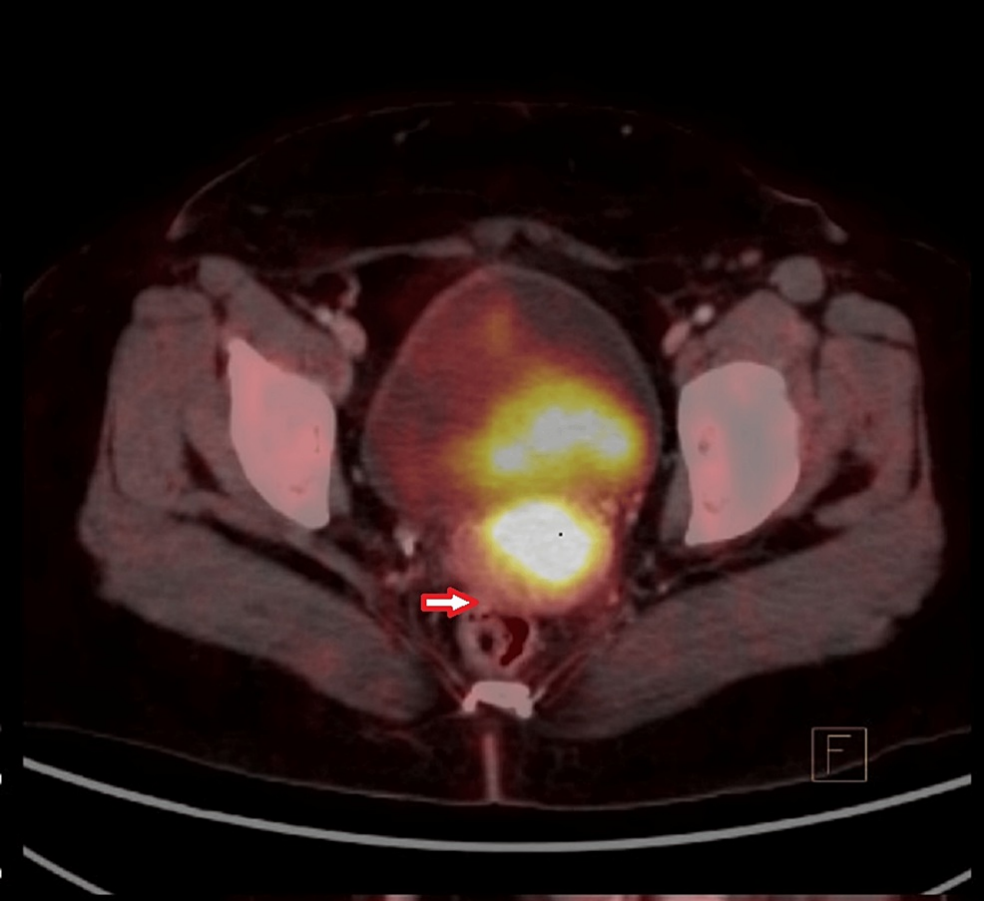
The PET/CT image showing involvement of parametrium The red arrow shows the extension of FDG uptake beyond the cervix. PET/CT: Positron emission tomography/computed tomography, FDG: Fluoro- D-glucose

If the extension of FDG uptake was beyond the cervix, then it was considered as positive parametrial involvement as shown in Figure 2.

**Figure 3:**
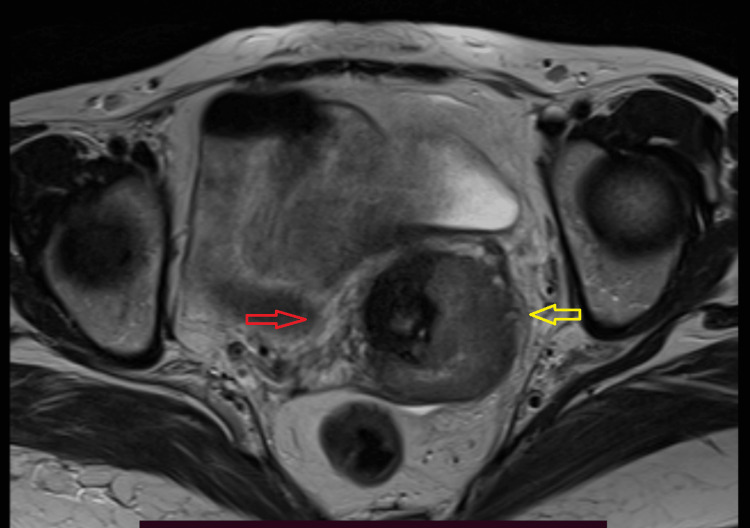
The MRI image showing involved and uninvolved parametrium in an early cervical cancer patient The yellow arrow shows an involved parametrium with loss of the hypointense ring. The red arrow shows an uninvolved parametrium with an intact hypointense ring.

The MRI was performed with a 1.5T MRI (Siemens Magnetom Aera 1.5T, Munich, Germany) with a phased array coil. The T2weighted(W)_sagittal, axial, T1W_sagittal, short TI inversion recovery (STIR) coronal and T1 with axial images were obtained with the field of view (FOV) 150 to 200mm, slice thickness 3.5mm, gap 1mm to 2mm sequences. The parametrial invasion was detected when there was disruption of the hypointense stromal ring with nodular or irregular tumor signal intensity extending to the parametrium and a smooth tumor-parametrial interface excludes parametrial invasion (Figure 3).

**Figure 4:**
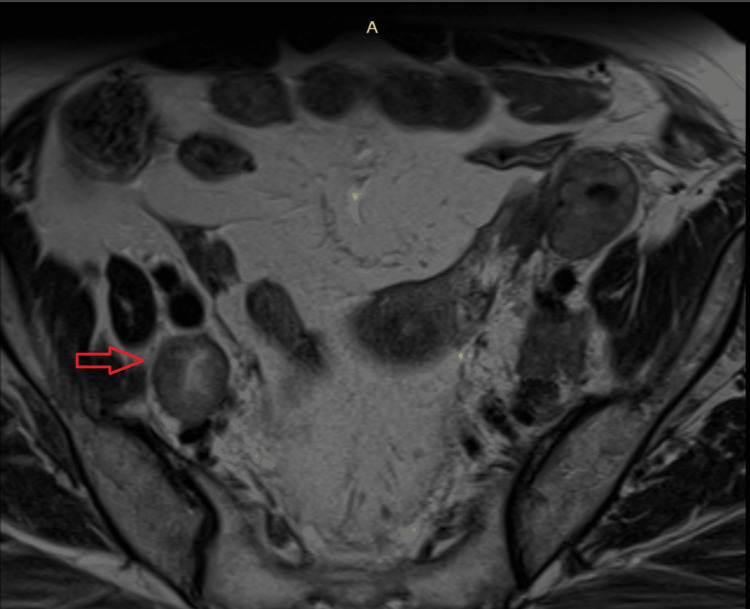
MRI image showing an involved right pelvic lymph node in an early cervical cancer patient The red arrow shows an enlarged right pelvic lymph node with a size of more than 1 cm

Short axis diameter of lymph nodes greater than or equal to 1 cm was considered pathological and the presence of intra-nodal necrosis was considered a confirmatory finding (Figure 4).

Patient demographic data, FIGO stage (2009), MRI findings, PET/CT findings, histopathology findings regarding local extent, lymph nodes, and parametrial involvement were recorded. The up or down staging with PET/CT and MRI was noted.

The sample size was calculated using the nMaster 2.0 software (CMC Biostatistics, Vellore, Tamil Nadu, India) based on the sensitivity of PET/CT (82%) and MRI (56%) from the study by Shi et al. [5]. Considering an alpha error of 0.05 and 80% power of the study, a sample size of 48 was estimated. Statistical analysis was done using R version 3.4.3 software [6]. Data were assessed for normality using the Shapiro-Wilk test. Categorical variables were expressed as proportions and analyzed using Fisher’s exact test or chi-square test as appropriate. Continuous variables were described as mean + standard deviation or median and range and analyzed using the Wilcoxon Rank Sum test for non-normally distributed data. Taking histopathological examination (HPE) report as the gold standard, sensitivity, specificity, positive predictive value (PPV), and negative predictive value (NPV) for each imaging modality for detection of parametrial and lymph nodal involvement were calculated along with 95% confidence intervals using standard statistical formulas. Exact binomial test, instead of McNemar’s test, was used to determine any statistically significant difference between the sensitivities and specificities of the two imaging modalities, when the number of patients with differing results for the two tests (discordant pairs) was small, i.e., <20. The relative predictive values method was used to determine any statistically significant difference between the PPV and NPV of the two imaging modalities. A p < 0.05 was considered significant for any test.

## Results

**Figure 5:**
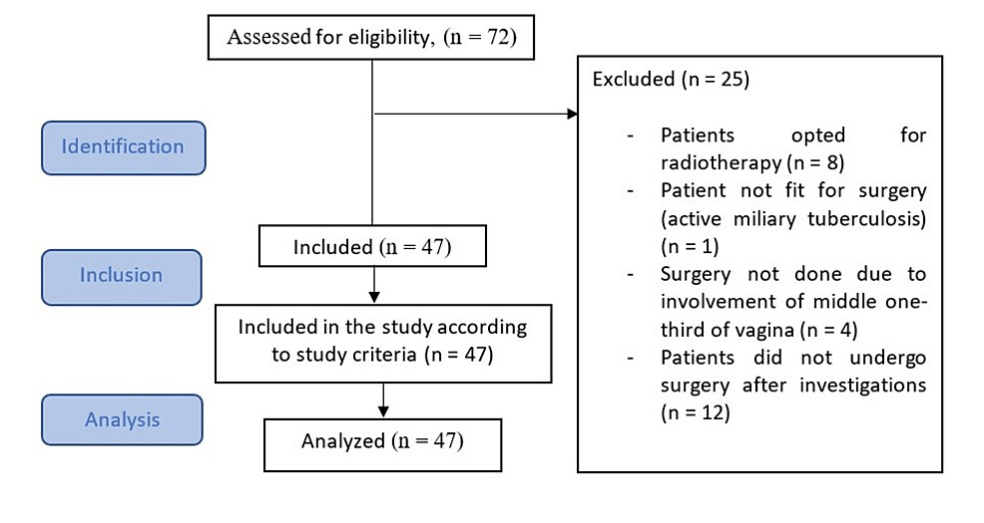
STROBE flow chart STROBE: Strengthening the Reporting of Observational Studies in Epidemiology

The present study reporting follows the Strengthening the Reporting of Observational Studies in Epidemiology statement as shown in Figure 5.

The mean age of the study population was 48.6 years. The most common histology was squamous cell carcinoma seen in 35 patients (74.45%) followed by adenocarcinoma in five patients (10.6%). The mean SUV of the tumor was 11.6. Most of the study population belonged to FIGO stage IB1. Table 1 summarizes the clinical, radiological, and pathological stage distribution of the study population.

**Table 1:** Clinical, radiological, and pathological stage distribution of the study population Data represented as frequency (n) and percentage (%). PET/CT: Positron emission tomography/computed tomography

FIGO Stage (2009)	Clinical stage n/total no. of patients (%)	MRI stage n/total no. of patients (%)	PET/CT stage n/total no. of patients (%)	Histopathological stage n/total no. of patients (%)
IA	2/47(4.2%)	8/47(17%)	4/47 (8.5%)	5/47(10.6%)
IB1	28/47(59.5%)	13/47(27.6%)	17/47(36.1%)	23/47( 48.9%)
IB2	2/47 (4.2%)	1/47(14.9%)	7/47(14.9%)	8/47(17%)
IIA1	13/47 ( 27.6%)	6/47(12.7%)	2/47(4.3%)	2/47(4.2%)
IIA2	2/47 (4.2%)	5/47(10.6%)	1/47(2%)	4/47(8.5%)
IIB	0/47(0%)	13/47(27.6%)	5/47(10.6%)	4/47(8.5%)
IV	0/47(0%)	1/47(2%)	11/47(23.4%)	0/47(0%)

A comparison of sensitivity, specificity, PPV, and NPV with confidence intervals of PET/CT and MRI for parametrial assessment is shown in Table 2.

**Table 2:** Comparison of diagnostic accuracy of MRI and PET/CT for parametrial assessment PET/CT: Positron emission tomography/computed tomography, PPV: Positive predictive value, NPV: Negative predictive value, CI: Confidence interval

Measures of diagnostic performance	MRI		PET/CT		p-value
	Estimate (%)	95% CI	Estimate (%)	95% CI	
Sensitivity	33.3	0-86.6	33.3	0-86.6	>0.9
Specificity	63.6	49.4-77.8	81.8	70.4-93.2	0.08
PPV	5.9	0-17	11.1	0-31.6	0.04
NPV	93.3	84.4-100	94.7	87.6-100	>0.9

In the present study, the sensitivity of MRI and PET/CT were similar for parametrial assessment. The PET/CT had higher specificity than MRI which is not statistically significant. The MRI had a significantly higher positive predictive value compared to PET/CT for parametrial assessment (p = 0.04).

A comparison of sensitivity, specificity, PPV, and NPV with confidence intervals of PET/CT and MRI for lymph nodal assessment is shown in Table 3.

**Table 3:** Comparison of diagnostic accuracy of MRI and PET/CT for lymph nodal assessment PET/CT: Positron emission tomography/computed tomography, PPV: Positive predictive value, NPV: Negative predictive value, CI: Confidence interval

Measures of diagnostic performance	MRI		PET/CT		p-value
	Estimate (%)	95% CI	Estimate (%)	95% CI	
Sensitivity	75	32.5-100	75	32.5-100	1
Specificity	81.3	69.7-93	83.7	72.6-94.7	1
PPV	27.2	0.9-53	30	1.5-58	0.8
NPV	97.2	91.8-100	97.2	92.1-100	0.9

In the present study, the sensitivity of MRI and PET/CT were similar for lymph nodal assessment. There was no significant difference concerning specificity, PPV, or NPV of MRI and PET/CT for lymph nodal assessment. Sensitivity, specificity, PPV, and NPV of MRI for assessment of vaginal involvement were 50%, 80%, 10%, and 97.2%, respectively. The corresponding values of PET/CT were 0%, 82.2%, 0%, and 100%, respectively. Sensitivity, specificity, PPV, and NPV of MRI for assessment of bladder and rectal involvement were 0%, 97.8%, 0%, and 100%, respectively. The corresponding values of PET/CT were 0%, 76.6%, 0%, and 100%, respectively.

The percentage of stage migration with MRI and PET/CT is shown in Table 4. There is no statistically significant difference in stage migration with MRI and PET/CT in comparison with final HPE.

**Table 4:** Comparison of stage migration with MRI and PET/CT Data represented as frequency (n) and percentage (%) PET/CT: Positron emission tomography/computed tomography

Imaging modality	Stage migration			p-value
	Down staging n (%)	No change in stage n (%)	Upstaging n (%)	
MRI	9 (19.1%)	14 (29.7%)	24 (51.1%)	0.4
PET/CT	8 (17.02%)	20 (42.5%)	19 (40.4%)	

## Discussion

In the present study, the sensitivity and specificity of MRI for parametrial assessment were 33% and 63.6%, respectively. In previous studies, the reported range of sensitivity and specificity of MRI for parametrial assessment was 62% to 81% and 77.5% to 88%, respectively [7-9]. Compared to previous studies, sensitivity and specificity were low in the present study [7-9]. The probable reasons for low sensitivity and specificity in the present study could be due to not using additional features of MRI (dynamic contrast enhancement (DCE)/diffusion-weighted imaging (DWI)/fat suppression). Also, parametrial fat stranding was considered as parametrial involvement which can be due to inflammation or infection, or compression effect. In the present study, the NPV of MRI for parametrial assessment was 93.3%. In previous studies, NPV was in the range of 53% to 95.9% [7-9]. High NPV infers that negative parametrium on MRI image has a high probability of negative parametrial margin on final histopathology.

There has been no prospective study to date that presents the role of PET/CT compared to MRI for assessing the involvement of parametrium in early cervical cancer patients. In the present study, the specificity and NPV of PET/CT were 81.8% and 92.3%, respectively. In previous retrospective studies, the specificity and NPV were 88.6% and 100%, respectively [10-12].

In the present study, the sensitivity and NPV of MRI for lymph node assessment were 75% and 97.2%, respectively. In previous studies, the sensitivity and NPV of MRI for lymph node assessment were reported in the range of 67% to 83% and 91% to 95%, respectively [13-15].

In the present study, the sensitivity and NPV of PET/CT for lymph node assessment were 75% and 97.3%, respectively. In previous studies, the sensitivity of PET/CT for lymph node assessment was in the range of 33% to 91% [13-15]. A previous retrospective study has shown an NPV of 98% [15]. Like previous studies, the present study had shown high NPV, which means that there was a high probability of lymph nodes being negative on final histopathology.

In the present study, MRI had 29.7% agreement with pathological staging. Around 51% were upstaged by MRI in comparison to histopathology which could be due to considering parametrial fat stranding as parametrial involvement which on final histopathology has shown negative parametrial involvement. About 19.1% were downstaged by MRI in reference to final histopathology. This could be due to the high percentage of nonvisible tumors on MRI. In previous studies, the downstaging and upstaging with MRI were 9.4% and 6.3%, respectively [16].

In the present study, PET/CT has 40.4% agreement with final histopathology, 17% were downstaged, and 42.7% were upstaged in reference to final histopathology. This high percentage of upstaging was due to false positive reporting of bladder and rectal involvement for which PET/CT was not used and in early cervical cancer, it is unlikely for the tumor to involve the bladder and or rectum.

In a study done by Kitajima et al., the sensitivity of MRI was 100% for the assessment of vaginal involvement which was higher than the present study (50%), but the specificity of MRI (96.3%) was comparable with the present study (97.2%) [14]. The sensitivity and specificity of PET/CT in the present study 0% and 82.2%, respectively, were lower than the sensitivity and specificity in the study by Kitajima et al.( 33.3% and 100%, respectively) [14]. These results show that MRI is a better imaging modality in the assessment of vaginal involvement by tumors.

Limitations of the present study include not using contrast, not using additional features like DWI, DCE, and fat-suppressed images of MRI, and not following proper intervals between biopsy and imaging. We recommend further prospective studies to confirm our findings and to determine the single best imaging modality for early cervical cancer staging.

## Conclusions

The PET/CT had no additional utility (compared to MRI) in the evaluation of local staging of clinically early cervical carcinoma patients. Given the low prevalence of lymph node involvement in early cervical cancer, and comparable diagnostic accuracy of PET/CT and MRI for lymph node assessment, MRI with its high spatial resolution can be used solely to assess the local extent and lymph node involvement, especially in a resource setting where PET/CT is not available.

## References

[1] Bray F, Ferlay J, Soerjomataram I, Siegel RL, Torre LA, Jemal A (2018). Global cancer statistics 2018: GLOBOCAN estimates of incidence and mortality worldwide for 36 cancers in 185 countries. CA Cancer J Clin.

[2] Grueneisen J, Schaarschmidt BM, Heubner M (2015). Integrated PET/MRI for whole-body staging of patients with primary cervical cancer: preliminary results. Eur J Nucl Med Mol Imaging.

[3] Yeh LS, Hung YC, Shen YY (2002). Detecting para-aortic lymph nodal metastasis by positron emission tomography of 18F-fluorodeoxyglucose in advanced cervical cancer with negative magnetic resonance imaging findings.. Oncol Rep.

[4] Sala E, Rockall AG, Freeman SJ, Mitchell DG, Reinhold C (2013). The added role of MR imaging in treatment stratification of patients with gynecologic malignancies: what the radiologist needs to know. Radiology.

[5] Runjun Shi, Jie Chen, Jinchang Wu (2016). PET/CT and MRI in evaluating cervical cancer. J Can Res Updates.

[6] (2019). R: The R Project for Statistical Computing. http://www.R-project.org.

[7] Roh HJ, Kim KB, Lee JH, Kim HJ, Kwon YS, Lee SH (2018). Early cervical cancer: predictive relevance of preoperative 3-Tesla multiparametric magnetic resonance imaging. Int J Surg Oncol.

[8] Park JJ, Kim CK, Park SY, Park BK, Kim B (2014). Value of diffusion-weighted imaging in predicting parametrial invasion in stage IA2-IIA cervical cancer. Eur Radiol.

[9] Qu JR, Qin L, Li X (2018). Predicting parametrial invasion in cervical carcinoma (stages IB1, IB2, and IIA):Diagnostic accuracy of T2-weighted imaging combined with DWI at 3 T. AJR Am J Roentgenol.

[10] Signorelli M, Guerra L, Montanelli L (2011). Preoperative staging of cervical cancer: is 18-FDG-PET/CT really effective in patients with early stage disease?. Gynecol Oncol.

[11] Dong Y, Wang X, Wang Y, Liu Y, Zhang J, Qian W, Wu S (2014). Validity of 18F-fluorodeoxyglucose positron emission tomography/computed tomography for pretreatment evaluation of patients with cervical carcinoma: a retrospective pathology-matched study. Int J Gynecol Cancer.

[12] Pandharipande PV, Choy G, del Carmen MG, Gazelle GS, Russell AH, Lee SI (2009). MRI and PET/CT for triaging stage IB clinically operable cervical cancer to appropriate therapy: decision analysis to assess patient outcomes. AJR Am J Roentgenol.

[13] Monteil J, Maubon A, Leobon S (2011). Lymph node assessment with (18)F-FDG-PET and MRI in uterine cervical cancer. Anticancer Res.

[14] Kitajima K, Suenaga Y, Ueno Y (2014). Fusion of PET and MRI for staging of uterine cervical cancer: comparison with contrast-enhanced (18)F-FDG PET/CT and pelvic MRI. Clin Imaging.

[15] Lv K, Guo HM, Lu YJ, Wu ZX, Zhang K, Han JK (2014). Role of 18F-FDG PET/CT in detecting pelvic lymph-node metastases in patients with early-stage uterine cervical cancer: comparison with MRI findings. Nucl Med Commun.

[16] Park W, Park YJ, Huh SJ (2005). The usefulness of MRI and PET imaging for the detection of parametrial involvement and lymph node metastasis in patients with cervical cancer. Jpn J Clin Oncol.

